# Laborchemisches und kalorimetrisches Monitoring der medizinischen Ernährungstherapie auf der Intensiv- und Intermediate Care Station: Zweites Positionspapier der Sektion Metabolismus und Ernährung der Deutschen Interdisziplinären Vereinigung für Intensiv- und Notfallmedizin (DIVI)

**DOI:** 10.1007/s00063-023-01001-2

**Published:** 2023-04-17

**Authors:** Gunnar Elke, Wolfgang H. Hartl, Michael Adolph, Matthias Angstwurm, Frank M. Brunkhorst, Andreas Edel, Geraldine de Heer, Thomas W. Felbinger, Christiane Goeters, Aileen Hill, K. Georg Kreymann, Konstantin Mayer, Johann Ockenga, Sirak Petros, Andreas Rümelin, Stefan J. Schaller, Andrea Schneider, Christian Stoppe, Arved Weimann

**Affiliations:** 1 Klinik für Anästhesiologie und Operative Intensivmedizin, Universitätsklinikum Schleswig-Holstein, Campus Kiel, Arnold-Heller-Straße 3 Haus R3, 24105 Kiel, Deutschland; 2 Klinik für Allgemein‑, Viszeral- und Transplantationschirurgie, Ludwig-Maximilians-Universität München – Klinikum der Universität, Campus Großhadern, München, Deutschland; 3 Universitätsklinik für Anästhesiologie und Intensivmedizin und Stabsstelle Ernährungsmanagement, Universitätsklinikum Tübingen, Tübingen, Deutschland; 4 Medizinische Klinik und Poliklinik IV, Ludwig-Maximilians-Universität München – Klinikum der Universität, Campus Innenstadt, München, Deutschland; 5 Zentrum für Klinische Studien, Klinik für Anästhesiologie und Intensivtherapie, Universitätsklinikum Jena, Jena, Deutschland; 6 Klinik für Anästhesiologie mit Schwerpunkt operative Intensivmedizin (CVK, CCM), Charité – Universitätsmedizin Berlin, Berlin, Deutschland; 7 Zentrum für Anästhesiologie und Intensivmedizin, Klinik für Intensivmedizin, Universitätsklinikum Hamburg-Eppendorf, Hamburg, Deutschland; 8 Klinik für Anästhesiologie, Operative Intensivmedizin und Schmerztherapie, Kliniken Harlaching und Neuperlach, Städtisches Klinikum München GmbH, München, Deutschland; 9 Klinik für Anästhesiologie, operative Intensivmedizin und Schmerztherapie, Universitätsklinikum Münster, Münster, Deutschland; 10 Kliniken für Anästhesiologie und Operative Intensivmedizin und Intermediate Care, Uniklinik RWTH Aachen, Aachen, Deutschland; 11 Flemingstraße 2, 22299 Hamburg, Deutschland; 12 Klinik für Pneumologie und Schlafmedizin, St. Vincentius-Kliniken, Karlsruhe, Deutschland; 13 Medizinische Klinik II, Klinikum Bremen Mitte, Bremen, Deutschland; 14 Interdisziplinäre Internistische Intensivmedizin, Universitätsklinikum Leipzig, Leipzig, Deutschland; 15 Anästhesie, Intensivmedizin und Notfallmedizin, Helios St. Elisabeth-Krankenhaus Bad Kissingen, Kissingen, Deutschland; 16 Medizinische Fakultät, Klinik für Anästhesiologie und Intensivmedizin, Technische Universität München, München, Deutschland; 17 Klinik für Gastroenterologie, Hepatologie und Endokrinologie, Medizinische Hochschule Hannover, Hannover, Deutschland; 18 Klinik und Poliklinik für Anästhesiologie, Intensivmedizin, Notfallmedizin und Schmerztherapie, Universitätsklinikum Würzburg, Würzburg, Deutschland; 19 Abteilung für Allgemein‑, Viszeral- und Onkologische Chirurgie, Klinikum St. Georg gGmbH, Leipzig, Deutschland

**Keywords:** Ernährung, Indirekte Kalorimetrie, Intensivmedizin, Kritische Erkrankung, Leitlinie, Nutrition, Indirect calorimetry, Intensive care medicine, Critical illness, Guideline

## Abstract

Dieses zweite Positionspapier der Sektion Metabolismus und Ernährung der Deutschen Interdisziplinären Vereinigung für Intensiv- und Notfallmedizin (DIVI) gibt Empfehlungen zum laborchemischen Monitoring der Makro- und Mikronährstoffzufuhr sowie zum Einsatz der indirekten Kalorimetrie im Rahmen der medizinischen Ernährungstherapie erwachsener Intensivpatient:innen. Zusätzlich werden Empfehlungen zur krankheitsbezogenen bzw. individuellen (Spiegelbestimmung) Substitution und (Hochdosis‑)Pharmakotherapie von Vitaminen und Spurenelementen vorgenommen.

## Abstract (en)

This second position paper of the Section Metabolism and Nutrition of the German Interdisciplinary Association for Intensive Care and Emergency Medicine (DIVI) provides recommendations on the laboratory monitoring of macro- and micronutrient intake as well as the use of indirect calorimetry in the context of medical nutrition therapy of critically ill adult patients. In addition, recommendations are given for disease-related or individual (level determination) substitution and (high-dose) pharmacotherapy of vitamins and trace elements.

**Graphic abstract:**

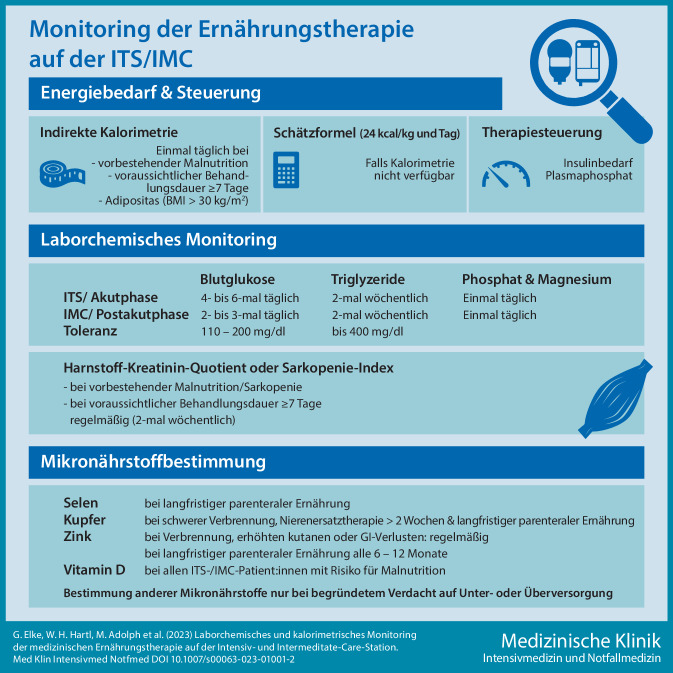

## 1. Präambel und Ziel

Im ersten Teil des Positionspapiers der Deutschen Interdisziplinären Vereinigung für Intensiv- und Notfallmedizin (DIVI) wurden konsensbasierte strukturierte Empfehlungen zur Erfassung und zum apparativem Monitoring des Ernährungsstatus von Patient:innen auf der Intensivstation (ITS) und Intermediate Care (IMC) Station gegeben [1]. Der zweite Teil ergänzt nun diese Empfehlungen um das laborchemische Monitoring der Makro- und Mikronährstoffzufuhr sowie um den Einsatz der indirekten Kalorimetrie im Rahmen der medizinischen Ernährungstherapie („medical nutrition therapy“, MNT). Ziel beider Positionspapiere ist es, im Sinne der DIVI-Mission 2030, und in Ergänzung zur aktuellen S2k-Leitlinie „Klinische Ernährung in der Intensivmedizin“ der Deutschen Gesellschaft für Ernährungsmedizin (DGEM) aus dem Jahr 2018 [2], Kompetenz und Qualität in der klinischen Durchführung der MNT weiterzuentwickeln. Auch in diesem zweiten Positionspapier werden ITS und IMC entsprechend der DIVI-Strukturempfehlungen definiert [3, 4], wobei die Empfehlungen zur Struktur und Ausstattung von Intensivstationen 2022 aktualisiert wurden [3]. In Bezug auf die Ernährungstherapie wird in diesen neuen Strukturempfehlungen empfohlen, dass „eine Mitarbeiter:in mit einer ernährungsmedizinischen Qualifikation mindestens arbeitstäglich zur Verfügung stehen sollte (Empfehlungsgrad 2C) und eine Mitbetreuung durch Ernährungsmediziner:innen oder Ernährungsfachkräfte bei speziellen Problemen, insbesondere in den höheren Versorgungsstufen 2 und 3, verfügbar sein sollte“ (Empfehlungsgrad 2C).

## 2. Methodisches Vorgehen und Konsensfindung

Im Rahmen eines Onlinesymposiums der DGEM in Zusammenarbeit mit der Sektion 4.3 Metabolismus und Ernährung der DIVI am 20./21.11.2020 wurde der Themenkomplex definiert und anhand der Präsentationsinhalte gemeinsam ein erster Textentwurf erstellt. Zwischenzeitlich erfolgte eine aktuelle Literatursuche anhand der auch für die Leitlinie verwendeten Schlüsselwörter und Suchstränge unter Einbeziehung nachfolgend publizierter Leitlinien. Der Textentwurf wurde den Sektionsmitgliedern am 31.01.2021 zur Durchsicht vorgelegt und nachfolgend in einer Onlinesitzung am 15.02.2021 strukturiert diskutiert. Dabei wurde festgelegt, den geplanten Inhalt auf 2 Positionspapiere aufzuteilen. Nach Publikation des ersten Positionspapiers folgten analog weitere Textüberarbeitungen dieses zweiten Positionspapiers im E‑Mail-Umlauf. Die abschließende Diskussion und Konsensusfindung erfolgte am 22.08.2022 online. Für die Empfehlungen bestand jeweils 100 %ige Zustimmung. Der überarbeitete Text wurde am 12.10.2022 zur finalen Durchsicht an die Sektionsmitglieder versandt. Am 17.11.2022 wurde die finale Version dem Präsidium der DIVI übermittelt und am 01.12.2022 verabschiedet.

## 3. Laborchemisches Monitoring des Ernährungsstatus

Die Bestimmung der Konzentrationen der hepatisch synthetisierten Proteine Albumin und Präalbumin im Serum gilt als „klassisch“ bei der Erhebung des Ernährungsstatus und ist außerhalb des intensivmedizinischen Kontextes in einigen Screeninginstrumenten ein weiterhin propagiertes Kriterium zur Erfassung einer Mangelernährung [5, 6]. Ein Zusammenhang besteht zwischen Inflammation und Malnutrition, jedoch nicht zwischen Malnutrition und viszeralem Proteingehalt. Bei kritisch kranken Patient:innen werden nach heutigem Verständnis Albumin- und Präalbuminkonzentrationen jedoch eher als Marker der Inflammation und des Ausmaßes der Homöostasestörung (bzw. des „capillary leak“) und nicht der Mangelernährung angesehen (vergleichbar mit dem Phasenwinkel der Bioimpedanzanalyse; [7]). Deswegen werden Albumin- und Präalbuminkonzentration auch nicht in der aktuellen DGEM-Definition der Mangelernährung berücksichtigt [8] und dienen – zusammen mit spezifischen Inflammationsmarkern – laut der Global Initiative of Malnutrition (GLIM) nur noch zur Diskriminierung des Schweregrads der Inflammation [5].

### Empfehlung

Die Bestimmung der Albumin- und Präalbuminkonzentration im Serum bei Aufnahme kann zur Beurteilung des Ernährungsstatus nicht empfohlen werden.

## 4. Laborchemisches Monitoring der MNT

### 4.1 Phosphat

Eine ausreichende Phosphatversorgung ist zentrale Voraussetzung für eine normale Zellfunktion und eine Hypophosphatämie sollte – auch wenn spezifische Studien für kritisch Kranke fehlen – immer eine Substitution nach sich ziehen [9]. Gemäß der Empfehlung der DGEM-Leitlinie [2] und insbesondere basierend auf den Ergebnissen einer randomisierten multizentrischen Studie [10] sollte bei Auftreten einer Hypophosphatämie (< 0,65 mmol/l) als Surrogatmarker eines Refeedingsyndroms unter MNT – und Ausschluss anderer möglicher Ursachen – eine Reduktion der vorbestehenden Makronährstoffzufuhr (Kalorien und Proteine) erfolgen. Schwere Hypophosphatämien werden regelmäßig bei Patient:innen z. B. nach großen Operationen insbesondere herzchirurgischen Eingriffen, diabetischen Entgleisungen (Ausgleich Ketoacidose) oder pharmakologisch induziert (z. B. Diuretika) beobachtet [11–14]. Allerdings ist die Steuerung der Makronährstoffzufuhr anhand des Phosphatspiegels bei Patient:innen mit Nierenersatztherapie nicht möglich.

Die Serumphosphatkonzentration sollte somit in der Akutphase der kritischen Erkrankung sowie bei Verdacht auf Refeedingsyndrom ab Beginn der MNT einmal pro Tag bestimmt werden. Erst bei nicht mehr substitutionspflichtigen Phosphatkonzentrationen sollte die Makronährstoffzufuhr schrittweise täglich wieder gesteigert werden [2]. Das Messintervall kann dann verlängert werden, z. B. auf 2‑mal wöchentlich, wenn die metabolische Situation der Patient:innen stabil ist (z. B. nach erfolgreicher Fokussanierung) und keine engmaschigere Bestimmung mehr aufgrund anderer Indikationen (mechanische Nierenersatztherapie) als notwendig erachtet wird.

### 4.2 Magnesium

Magnesium spielt eine wichtige Rolle bei der Kontrolle der Herzfrequenz, der Muskelkontraktion und -relaxation, der nervalen Reizübermittlung und des vaskulären Tonus. Aus diesem Grund sollte auch eine Hypomagnesiämie korrigiert werden [15]. Eine Hypomagnesiämie kann auch im Zusammenhang mit einem Refeedingsyndrom auftreten und insbesondere einen paralytischen Ileus bzw. Herzrhythmusstörungen auslösen oder aggravieren. Andere Ursachen können speziell bei ITS-/IMC-Patient:innen intestinale oder renale Verluste bzw. endokrine oder pharmakologische Nebenwirkungen sein. Auch eine Malnutrition bzw. eine chronisch ungenügende Magnesiumzufuhr können zu einer Hypomagnesiämie führen. Magnesium sollte bei einem Risiko für ein Refeedingsyndrom, insbesondere wenn die Phophatkonzentration z. B. aufgrund eines Nierenersatzverfahrens nicht aussagekräftig ist, einmal täglich bestimmt werden. Auch hier kann das Messintervall dann verlängert werden, z. B. auf 2‑mal wöchentlich, wenn die metabolische Situation der Patient:innen stabil ist und generell keine engmaschigere Bestimmung aufgrund anderer, z. B. endokrinologischer oder kardiologischer Indikationen als notwendig erachtet wird.

### 4.3 Blutzucker

Bezüglich der anzustrebenden Konzentrationen lauten die älteren Empfehlungen der DGEM aus dem Jahr 2013 [16]: „Bei kritisch Erkrankten können Blutzuckerkonzentrationen zwischen 140–200 mg/dl (7,7–11,0 mmol/l) toleriert werden und es soll ein Zielwert von 110 mg/dl (6,1 mmol/l) nicht unterschritten werden.“ Eine Individualisierung nach der Ausgangs-HbA1c-Konzentration hat bisher keinen Vorteil gezeigt [17], bei Diabetes Typ II können jedoch nach neuesten Erkenntnissen möglicherweise auch höhere Konzentrationen (< 250 mg/dl bzw. < 13,9 mmol/l) toleriert werden [18, 19].

Die Steuerung der Insulintherapie bzw. MNT in Abhängigkeit der Blutzuckerkonzentrationen ist in Kap. 6 aufgeführt. Auf der ITS sollte die Glukosekonzentration in der Akutphase 4‑ bis 6‑mal pro Tag gemessen werden, in der Postakutphase und im IMC-Bereich 2‑ bis 3‑mal pro Tag, sofern keine engmaschigere Bestimmung aufgrund anderer Indikationen (hoher Insulinbedarf, schwere Hyper‑/Hypoglykämie) notwendig ist.

### 4.4 Triglyzeride

Hypertriglyzeridämien können bei Patient:innen auf der ITS/IMC mit Sepsis (defiziente Triglyzeridlipase) in Kombination mit einer Sedierung mittels Propofol, einer Zufuhr fetthaltiger Nährlösungen und/oder einer relativen Überernährung mit Kohlenhydraten auftreten. Eine (auch erworbene) Hypertriglyzeridämie ist immer Ausdruck einer Fettverwertungsstörung im Rahmen der VLDL_1_-VLDL_2_-IDL-LDL-Kaskade und die Hypertriglyzeridämie stellt einen relevanten Risikofaktor für eine akute Pankreatitis dar. Neuere Untersuchungen deuten darauf hin, dass ein signifikanter, wenn auch absolut nur geringer Anstieg des Risikos für eine Pankreatitis bereits zwischen 177 und 265 mg/dl bzw. 2,0 und 3,0 mmol/l zu beobachten ist [20, 21]. Darüber hinaus scheint eine Assoziation der Hypertriglyzeridämie mit der Entwicklung eines chronischen Nierenversagens zu bestehen [22, 23]. In kleineren (methodisch limitierten) Beobachtungsstudien konnte jedoch bei bis zu 440 mg/dl (5 mmol/l) keine Assoziation mit einem schlechteren Outcome gefunden werden [24]. Die ESPEN empfiehlt für kritisch Kranke aktuell, ab Konzentrationen > 500 mg/dl (5,6 mmol/l) eine Ursachenforschung bez. der Pathomechanismen (Hyperalimentation) einzuleiten [25], während die A.S.P.E.N. gegenwärtig einen oberen Grenzwert von 400 mg/dl (4,5 mmol/l) empfiehlt [26]. Die älteren DGEM-Empfehlungen hierzu lauten [16]: „In Akutsituationen (auch bei kritisch kranken Patient:innen) können Triglyzeridkonzentrationen bis zu 400 mg/dl (4,6 mmol/l) toleriert werden“. Wir würden diese Empfehlungen dahingehend modifizieren, dass bei kritisch Kranken mit akuter Pankreatitis nur noch Triglyzeridkonzentrationen bis zu 250 mg/dl (2,8 mmol/l) toleriert werden sollten.

Im Fall einer Hypertriglyzeridämie wird eine Reduktion der (parenteralen) Fettzufuhr solange empfohlen, bis wieder Konzentrationen < 400 mg/dl (< 4,6 mmol/l) bzw. < 250 mg/dl (< 2,8 mmol/l) bei Pankreatitis erreicht werden. Das Minimum einer ein- bis 2‑maligen Fettzufuhr/Woche sollte jedoch auf Dauer nicht unterschritten werden, um einem Mangel an essenziellen Fettsäuren vorzubeugen. Bei anhaltend hohen Triglyzeridspiegeln kann auch eine Reduktion der Kohlenhydratzufuhr sinnvoll sein. Auf der ITS/IMC sollte – in Anlehnung an das aktuelle ESPEN-Positionspapier „Monitoring nutrition in the ICU“ [25] – der Triglyzeridspiegel im Allgemeinen 2‑mal wöchentlich bestimmt werden; bei pathologischen Werten, speziellen Krankheitsbildern (akute Pankreatitis) und unter parenteraler Fettzufuhr (inklusive Sedierung mit Propofol) sind häufigere Kontrollen angezeigt (einmal pro Tag).

### 4.5 Serumharnstoff-Kreatinin-Quotient, Sarkopenie-Index und Harnstoff-Stickstoff-Verlust

Wie bereits im 1. Positionspapier beschrieben ist bei kritisch Kranken das laborchemische (und apparative) Monitoring der Muskelmasse in der klinischen Routine eingeschränkt [1]. Daher wird in der DGEM-Leitlinie im Wesentlichen auf die Therapiesteuerung anhand des Ruheenergieumsatzes (REU) und der metabolischen Toleranz mittels Insulinbedarf, Glukosekonzentration und Phosphat verwiesen (Kap. 6; [2]).

Sowohl der Harnstoff-Kreatinin-Quotient als auch der sog. Sarkopenie-Index können neben der Beurteilung der Nierenfunktion als Marker der Katabolie eingesetzt werden [27–33]. Erhöhte Plasmakreatininkonzentrationen sind fast immer eine Folge einer verminderten glomerulären Filtrationsrate (GFR) und haben daher eine renale Ursache. Erniedrigte oder normale Konzentrationen bei gleichzeitiger Nierendysfunktion deuten auf eine Abnahme der Muskelmasse hin. Erhöhte Harnstoffkonzentrationen sind in Abwesenheit einer renalen Dysfunktion Resultat einer erhöhten hepatischen Produktion durch ein hohes endogenes (Katabolie) oder exogenes (Protein‑/Aminosäurenzufuhr im Rahmen der MNT) Stickstoffangebot. Alternativ kann eine Nierenfunktionsstörung zu einer reduzierten renalen Harnstoffelimination und konsekutiven Konzentrationserhöhung führen [27–33].

Bedeutung des Harnstoff-Kreatinin-Quotienten (Normbereich bei Einheit mg/dl: 20–35):

- 20–35: keine Katabolie und normale GFR;
- < 20: verminderte Katabolie (Malnutrition, Leberdysfunktion), relativ zu niedrige Proteinzufuhr und/oder eingeschränkte GFR;
- > 35: verstärkte Katabolie oder geringe Muskelmasse, relativ zu hohe Proteinzufuhr oder verringerte renale Harnstoffelimination.

Eine zuverlässige Korrelation zwischen dem Harnstoff-Kreatinin-Quotienten und dem Ausmaß der Katabolie ist somit nur in Abwesenheit einer klinisch relevanten renalen Dysfunktion (AKI-Stadium 0) zu erwarten.

Um dieses Problem zu umgehen, kann der sog. Sarkopenie-Index berechnet werden als:

- Serumkreatinin × 100/Serum-Cystatin‑C.

Dieser Index bietet eine objektivere Alternative zur Vorhersage und ggf. zum Monitoring des Ausmaßes der Katabolie bzw. der Muskelmasse, auch in Verbindung mit den im ersten Positionspapier diskutierten bildgebenden Methoden [1]. Ein niedriger Sarkopenie-Index ist mit einer geringeren Muskelmasse assoziiert und kann auch – wenn bereits bei Aufnahme auf eine ITS/IMC gemessen – als prognostischer Marker eingesetzt werden. Erforderlich ist allerdings die Messung der Cystatin-C-Konzentration im Serum.

In der ESPEN-Leitlinie wird darüber hinaus bei adipösen Intensivpatient:innen die Steuerung der Proteinzufuhr anhand des Harnstoff-Stickstoff-Verlusts im Urin empfohlen [34]. Allerdings ist aufgrund des methodischen Aufwands in der klinischen Praxis und unsicherer Relevanz hinsichtlich des klinischen Effekts [35, 36] der Evidenzgrad für diese Empfehlung gering (Expertenmeinung der ESPEN-Leitlinien-Gruppe).

#### Empfehlung

- Auf der ITS/IMC sollte die Phosphat- und die Magnesiumkonzentration einmal täglich bestimmt werden, sofern keine engmaschigere Bestimmung aufgrund anderer Indikationen als notwendig erachtet wird.
- Auf der ITS sollte die Glukosekonzentration in der Akutphase 4‑ bis 6‑mal pro Tag gemessen werden, in der Postakutphase und auf der IMC 2‑ bis 3‑mal pro Tag, sofern keine engmaschigere Bestimmung aufgrund anderer Indikationen notwendig ist.
- Auf der ITS/IMC sollte in der Akutphase der Triglyzeridspiegel 2‑mal pro Woche gemessen werden, sofern keine engmaschigere (tägliche) Bestimmung aufgrund bereits erhöhter Triglyzeridspiegel, spezieller Grunderkrankungen (akute Pankreatitis) oder zusätzlicher parenteraler Fettzufuhr (inklusive Propofol) notwendig ist.
- Bei Patient:innen mit vorbestehender Malnutrition oder Sarkopenie, einer voraussichtlichen Behandlungsdauer ≥ 7 Tage, und in Abwesenheit einer klinisch relevanten renalen Dysfunktion, kann zur Verlaufsbeurteilung der Katabolie/Muskelmasse bzw. Steuerung der Proteinzufuhr der Harnstoff-Kreatinin-Quotient regelmäßig (z. B. 2‑mal/Woche) bestimmt werden. Bei Patient:innen mit renaler Dysfunktion kann ersatzweise der Sarkopenie-Index verwendet werden.

### 4.6 Mikronährstoffe

Mikronährstoffe umfassen Spurenelemente sowie wasser- und fettlösliche Vitamine, die in einzelnen Organen und Kompartimenten in sehr unterschiedlichen und präzise geregelten Konzentrationen mit unterschiedlichen Eigenschaften und Wirkmechanismen vorkommen.

Bei Gesunden ist relativ gut belegt, welche Mengen an Mikronährstoffen im Rahmen einer natürlichen Ernährung aufgenommen werden sollten, entsprechende Referenzwerte finden sich auf der Webseite der Deutschen Gesellschaft für Ernährung (DGE; [37]). Tab. 1 gibt eine Übersicht zu verschiedenen Richtwerten.

**Table 1:** 

Mikronährstoffe	Tagesbedarf für gesunde Erwachsene (Alter 31–70 Jahre)	Tageszufuhr unter bilanzierter EE^a^ gemäß EU-Richtlinie	Gehalt kommerzieller Präparate zur EE^b^	Gehalt kommerzieller Präparate zur PE
*Spurenelemente*				
Chrom	20–35 µg	18,75–225 µg	35–150 µg	10 µg/10 µg
Kupfer	0,9 mg	0,9–7,5 mg	1–3 mg	1,3 mg/0,4 mg
Fluorid	3–5 mg	0–3 mg	0–3 mg	0,95 mg/0,95 mg
Iod	150 µg	97,5–525 µg	150–300 µg	130 µg/130 µg
Eisen	8 mg	7,5–30 mg	18–30 mg	1,1 mg/1,1 mg
Mangan	1,8–2,3 mg	0,75–7,5 mg	2–3 mg	0,27 mg/–
Molybdän	45 µg	52,5–270 µg	50–250 µg	19 µg/19 µg
Selen	55 µg	37,5–150 µg	50–150 µg	32 µg/78 µg
Zink	8–11 mg	7,5–22,5 mg	10–20 mg	6,5 mg/5,0 mg
*Vitamine*				
A (Retinol)^c^	700–900 µg	525–2700 µg	900–1500 µg	1,05 mg/3530 IE
D (Cholecalciferol)	15–20 µg	7,5–37,5 µg	25 µg	55 µg/200 IE
E (α-Tocopherol)	15 mg	7,5–45 mg	15 mg	10,2 mg/10 IE
K (Phyllochinon)^d^	90–120 µg	52,5–300 µg	120 µg	–/150 µg
B_1_ (Thiamin)	1,1–1,2 mg	0,9–7,5 mg	1,5 mg^f^	3,51 mg/2,5 mg
B_2_ (Riboflavin)	1,1–1,3 mg	1,2–7,5 mg	1,2 mg^f^	4,14 mg/3,6 mg
B_3_ (Niacin)	11–16 mg	13,5–45 mg	18 mg^f^	46 mg/40 mg
B_5_ (Pantothensäure)	5 mg	2,25–22,5 mg	5 mg^f^	17,25 mg/15 mg
B_6_ (Pyridoxalphosphat)^e^	1,5–1,7 mg	1,2–7,5 mg	1,5 mg^f^	4,53 mg/4 mg
B_7_ (Biotin)	30 µg	11,25–112,5 µg	30 µg^f^	69 µg/60 µg
B_9_ (Folsäure)	400 µg	150–750 µg	330–400 µg^f^	–/400 µg
B_12_ (Cyanocobalamin)	2,4 µg	1,05–10,5 µg	>2,5 µg^f^	–/5 µg
C (Ascorbinsäure)	75–90 mg	33,75–330 mg	100 mg^f^	125 mg/100 mg

*EE* enterale Ernährung, *PE* parenterale Ernährung

Kobalt wird als Vitamin B_12_ bereitgestellt. Da es keine Angaben zum Tagesbedarf für Carnitin, Cholin und CoQ10 gibt, sind diese nicht in der Tabelle aufgeführt. Zum Erreichen einer Hochdosis einzelner Mikronährstoffe sollte auf die Verwendung von Einzelpräparationen zurückgegriffen werden

^a^Die EU-Richtlinie regelt den Inhalt von Lebensmitteln für besondere medizinische Zwecke, wobei die Mengen pro 100 kcal angegeben werden. Minimale und maximale Dosen entsprechend einer Zufuhr von 1500 kcal/Tag angegeben

^b^Bilanzierte Mikronährstoffmenge in enteralen Nährlösungen. Im Fall einer höheren Nährstoffzufuhr (z. B. 2000 kcal pro Tag oder mehr) stellt eine Überschreitung dieser Empfehlung kein Risiko dar, wenn man die oberen tolerierbaren Werte berücksichtigt

^c^Retinol umfasst Retinol und Retinylester

^d^Bei Verwendung einer kompletten PE wird der Vitamin-K-Bedarf in der Regel durch Lipidemulsionen gedeckt

^e^Unter der Bezeichnung Vitamin B6 werden verschiedene vitaminwirksame Verbindungen zusammengefasst, wie z. B. Pyridoxin, Pyridoxamin und Pyridoxal, deren aktiver Metabolit Pyridoxalphosphat ist

^f^Bei wasserlöslichen Vitaminen handelt es sich bei den empfohlenen Mengen um Mindestmengen; mehr kann in der Regel sicher zugeführt werden

Veränderungen des Mikronährstoffhaushalts resultieren bei kritisch kranken Patient:innen

- als direkte Folge der akuten Homöostasestörung;
- als Nebenwirkung bestimmter Pharmaka (z. B. Diuretika, Protonenpumpeninhibitoren);
- als indirekte Folge der Therapie mittels extrakorporaler Verfahren (kontinuierliche Nierenersatztherapie, extrakorporale Membranoxygenierung), die insbesondere mit Verlusten der wasserlöslichen Vitamine, der fettlöslichen Vitamine A und D sowie von Kupfer und Eisen einhergehen [39–44].

Darüber hinaus kann ein Mikronährstoffdefizit bereits unabhängig von der kritischen Erkrankung vorliegen, z. B. bei vorbestehender Mangelernährung.

Für kritisch kranke Patient:innen ist neben der rein prognostischen Relevanz niedriger Mikronährstoffspiegel die therapeutische Relevanz einer regelmäßigen Plasmaspiegelbestimmung für die meisten Mikronährstoffe (speziell Vitamine) bislang nicht eindeutig geklärt [38]. Die Bestimmung in den klinisch verfügbaren Materialien (meistens Vollblut, Serum oder Plasma) weist zudem methodische Schwierigkeiten auf (speziell intrazellulär, Ex-vivo-Degradierung von UV-/thermo-/pH-labilen Mikronährstoffen) bzw. sind entsprechende Verfahren in der klinischen Routine oft nicht verfügbar.

Aus sequenziellen Untersuchungen ist bekannt, dass die Konzentration der Mikronährstoffe im Plasma hohen Schwankungen unterliegt und sich innerhalb weniger Stunden sehr stark ändern kann. Die Akutphasereaktion der systemischen Inflammation kann die Plasmaspiegel von Mikronährstoffen im Sinne einer inflammatorisch getriggerten Umverteilungsreaktion relevant verändern, vermittelt dadurch, dass einige Vitamine und Spurenelemente nicht frei im Plasma sondern an Bindungsproteinen gekoppelt vorliegen und typischerweise diese Bindeproteine und zudem Albumin oft erniedrigt sind [45]. Darüber hinaus scheint die Messung von sog. Surrogatmarkern, wie beispielsweise des Selenoproteins oder der Glutathionperoxidaseaktivität, eher den Mikronährstoffstatus bzw. dessen biologische Aktivität widerzuspiegeln als die Konzentration des Spurenelements Selen im Vollblut und Plasma selbst [46].

Ebenso ungeklärt ist die Höhe der Mikronährstoffzufuhr bei kritisch Kranken, vor allem im Hinblick auf den Zeitpunkt des Beginns, auf die Dosierung und Dauer, und auf die Selektion geeigneter Patient:innen. Bei einem veränderten Mikronährstoffhaushalt sind aus klinischer Sicht also 4 Handlungsmuster denkbar:

- klinische Toleranz (Hinweis auf eine endogene vorteilhafte adaptive Reaktion) unter Ignoranz der Plasmakonzentration;
- Substitution (Korrektur von Mikronährstoffdefiziten) entweder individuell (nach Spiegelbestimmung bis zum Erreichen der normalen Konzentration) oder krankheitsbezogen (ohne Spiegelbestimmung unter der Annahme eines krankheitsbezogenen universellen Mangels);
- krankheitsbezogene oder individuelle (Hochdosis‑)Pharmakotherapie (Erreichen supranormaler Konzentrationen) mit dem zusätzlichen, jedoch bis heute weitgehend putativen Ziel einer Optimierung der Stoffwechselleistung (z. B. Reduktion von oxidativem Stress, Verbesserung der körpereigenen immunologischen Kompetenz) bzw. Prävention von Organdysfunktionen;
- Betrachtung als Indikatorvariable und Ableitung einer Handlungskonsequenz auf einem anderen Gebiet entsprechend der Mikronährstoff-Plasmakonzentration.

In Analogie zu Gesunden empfiehlt die DGEM-Leitlinie, dass Vitamine und Spurenelemente bei kritisch Kranken dann substituiert werden sollten, wenn mit einer enteralen Ernährung der Tagesbedarf Gesunder nicht gedeckt werden kann oder wenn eine (supplementäre) parenterale Ernährung notwendig ist (krankheitsbezogene Zufuhr ohne Individualisierung). Insbesondere eine parenterale Ernährung ohne Mikronährstoffe kann das Risiko einer unzureichenden Zufuhr erhöhen und soll somit immer die intravenöse Substitution von Spurenelement- und Multivitaminpräparaten beinhalten [2].

2022 wurde die ESPEN-Leitlinie „Mikronährstoffe“ [38] publiziert, in der sich detaillierte Informationen zu allen Mikronährstoffen und krankheitsbezogene Empfehlungen zu Diagnostik und Therapie finden. Tab. 2 stellt die Empfehlungen aus dieser Leitlinie für kritisch kranke Patient:innen den Empfehlungen zu Mikronährstoffen der DGEM-Leitlinie Intensivmedizin 2018 [2] gegenüber. Zusätzlich geben wir in dieser Tabelle unter Berücksichtigung beider Leitlinien und nachfolgend publizierter RCT und systematischer Reviews und Metaanalysen (SRMA) aktualisierte Vorschläge zu den DGEM-Empfehlungen.

**Table 2:** 

	DGEM-Leitlinie Intensivmedizin [2]	ESPEN-Leitlinie Mikronährstoffe [38]	Empfehlungen DIVI-Sektion
Vitamin B_1_			
Therapie	Eine Pharmakotherapie mit Thiamin kann bei klinischen Anhaltspunkten für Thiaminmangel (z. B. bei chronischem Alkoholabusus, Mangelernährung) durchgeführt werden	Bei kritisch Kranken sollten 100–300 mg/Tag i.v. ab Aufnahme für 3–4 Tage zugeführt werden	*Vorschlag Beibehaltung der DGEM-Empfehlung* 1 SRMA mit 8 RCT/10 Kohortenstudien: Thiaminmonotherapie mit geringerer Inzidenz delirassoziiert, kein Effekt auf Mortalität [47]
Laborkontrollen	Keine spezifische Empfehlung vorhanden	Bei Refeedingsyndrom und Enzephalopathie sollte der Thiaminstatus mittels Thiamindiphosphat(ThDP)-Bestimmung in Erythrozyten oder Vollblut ermittelt werden	*Änderungsvorschlag der DGEM-Empfehlung* Aufgrund hoher therapeutischer Breite von Thiamin erscheint die Messung verzichtbar. Eine Thiaminpharmakotherapie sollte demnach nach klinischen Anhaltspunkten erfolgen
Vitamin C			
Therapie	Eine Pharmakotherapie mit Zink, Vitamin A, C und E oder deren Kombination sollte nicht routinemäßig durchgeführt werden	Bei kritischer Erkrankung sollte eine höhere Dosis von 2–3 g pro Tag intravenös als Repletionsdosis in der Akutphase gegeben werden	*Vorschlag Beibehaltung der DGEM-Empfehlung* Eine aktuelle multizentrische RCT zur Hochdosis-Vitamin-C-Therapie bei Sepsis zeigte ein höheres Risiko für den Compositeendpunkt Versterben oder Organversagen an Tag 28 vs. Placebo [48], eine nachfolgende Metaanalyse ergab keinen Hinweis für ein besseres klinisches Outcome [49]
Laborkontrollen	Keine spezifische Empfehlung vorhanden	Nicht empfohlen, da Messergebnisse schwer interpretierbar	*Änderungsvorschlag der DGEM-Empfehlung:* Aufgrund der o. g. Ergebnisse zum Therapieeffekt von hochdosiertem Vitamin C sollte eine Vitamin-C-Spiegelbestimmung nicht durchgeführt werden
Vitamin D			
Therapie	Eine Pharmakotherapie mit Vitamin D bis zu 10.000 IE Vitamin D enteral kann bei schwerem Vitamin-D-Mangel (25[OH]D ≤ 30 nmol/l entsprechend ≤ 12 ng/ml) erfolgen	Vitamin D (4000–5000 IE [100–125 mg] pro Tag) sollte bei Mangel 2 Monate lang verabreicht werden, um Blutspiegel von 25(OH)D zwischen 40 und 60 ng/ml zu erreichen. Es können wesentlich höhere Dosen erforderlich sein. Der Schweregrad des Mangels und die für die Behandlung erforderliche Dosis bestimmen die Häufigkeit der Blutbestimmung für Wirksamkeit und Sicherheit. Keine spezifische Empfehlung für ITS/IMC	*Vorschlag Beibehaltung der DGEM-Empfehlung* In der aktuellsten SRMA (16 RCTs, *N* = 2449) war eine Vitamin-D-Supplementierung mit einer signifikant niedrigeren Mortalität, kürzeren Verweil- und Beatmungsdauer assoziiert, unabhängig vom Baseline-Vitamin-D-Status [50]
Laborkontrollen	Ja, aber keine Empfehlung zu Indikation, Zeitpunkt und Frequenz der Messung	Vitamin-D-Konzentration (Serum 25(OH)D) kann bei allen Patient:innen mit Risiko für Mangelernährung oder Vitamin-D-Mangel bestimmt werden	*Änderungsvorschlag der DGEM-Empfehlung:* Übernahme der Empfehlung entsprechend der ESPEN-Leitlinie Mikronährstoffe
Kupfer (Cu)			
Therapie	Keine spezifische Empfehlung vorhanden	Individualisierte Substitution bei – schweren Verbrennungen – CRRT > 2 Wochen – langfristiger PE	*Änderungsvorschlag der DGEM-Empfehlung:* Übernahme der Empfehlung entsprechend der ESPEN-Leitlinie Mikronährstoffe In einer aktuellsten SRMA (37 RCT, *N* = 4905) wurde eine Kupfersupplementierung nicht als Mono-, sondern nur Kombinationstherapie mit Selen und Zink untersucht und war hier mit einer signifikant geringeren Verweildauer auf der ITS und im Krankenhaus assoziiert [51]
		Bei Plasma-Cu < 12 mmol/l kann eine Kupfer-Gabe erwogen werden. Bei Plasma-Cu < 8 mmol/l sollte Kupfer in einer Dosierung von 4–8 mg/Tag als intravenöse Kurzinfusion appliziert werden	
Laborkontrollen	Keine spezifische Empfehlung vorhanden	Der Plasma-Cu-Spiegel sollte bestimmt werden bei Patient:innen mit – schweren Verbrennungen – CRRT > 2 Wochen – langfristiger PE	*Änderungsvorschlag der DGEM-Empfehlung:* Übernahme der Empfehlung entsprechend der ESPEN-Leitlinie Mikronährstoffe
Selen (Se)			
Therapie	Eine Pharmakotherapie mit Selen soll nicht durchgeführt werden	Krankheits-bezogene Substitution bei RRT und Verbrennungen (375 µg/Tag)	*Änderungsvorschlag der DGEM-Empfehlung:* Übernahme der Empfehlung entsprechend ESPEN-Leitlinie Mikronährstoffe Eine krankheitsbezogene hochdosierte Pharmakotherapie (1000–4000 µg/Tag) kann weiterhin nicht empfohlen werden
		Individualisierte Substitution unter Langzeit-PE (> 2 Wochen); bei Plasma-Se < 0,4 mmol/l (< 32 µg/l) sollte Se 100 mg/Tag (i.v.) bis Normalisierung gegeben werden	
		Krankheits-bezogene Pharmakotherapie nach Polytrauma/herzchirurgischen Eingriffen (275 µg/Tag)	
Laborkontrollen	Keine spezifische Empfehlung vorhanden	Der Selenspiegel sollte bei Langzeit-PE (> 2 Wochen) bestimmt werden. Zur Bestimmung des Status ist die Selen-Konzentration im Plasma erforderlich, idealerweise sollte GPX‑3 im Plasma bestimmt werden (funktioneller Status). Die gleichzeitige Bestimmung von CRP und Albumin ist für die Auswertung erforderlich	*Änderungsvorschlag der DGEM-Empfehlung:* Übernahme der Empfehlung entsprechend ESPEN-Leitlinie Mikronährstoffe
Zink (Zn)			
*Pharmakotherapie*	Eine Pharmakotherapie mit Zink, Vitamin A, C und E oder deren Kombination sollte nicht routinemäßig durchgeführt werden	Individualisierte Substitution bei – Verbrennungen > 20 % Körperoberfläche; es sollten 30–35 mg/Tag i.v. für 2–3 Wochen verabreicht werden – längerfristiger PE und GI-Verlusten; es können bis zu 12 mg/Tag i.v. für die Dauer der Verluste/PE verabreicht werden	*Änderungsvorschlag der DGEM-Empfehlung:* Übernahme der Empfehlung entsprechend ESPEN-Leitlinie Mikronährstoffe
Laborkontrollen	Keine spezifische Empfehlung vorhanden	Bei Patient:innen mit erhöhten GI-Verlusten/Verbrennungen sollte der Plasma-Zn-Spiegel regelmäßig, bei langfristiger PE alle 6–12 Monate bestimmt werden	*Änderungsvorschlag der DGEM-Empfehlung:* Übernahme der Empfehlung entsprechend ESPEN-Leitlinie Mikronährstoffe

*AOX* Antioxidanzien, *CRRT* kontinuierliche Nierenersatztherapie, *DGEM* Deutsche Gesellschaft für Ernährungsmedizin, *GI* gastrointestinal, *i.v.* intravenös, *PE* parenterale Ernährung, *SRMA* systematischer Review und Metaanalyse, *GPX* Glutathionperoxidase

### 4.7 Glutamin

Die Aminosäure Glutamin erfüllt grundlegende Funktionen im Intermediärstoffwechsel als Transportmolekül für andere Aminosäuren im „Interorgansubstratfluss“, als Vorstufe für verschiedene Biosyntheseprozesse einschließlich der Immunantwort und als Energiesubstrat für Zellen mit hoher proliferativer Aktivität wie Darmepithelzellen. Sowohl niedrige (< 400 µmol/l) als auch sehr hohe Glutaminspiegel (> 930 µmol/l) im Serum haben konkordant zu Mikronährstoffspiegeln prinzipiell eine prognostische Relevanz [52]. Eine Pharmakotherapie mit Glutamin wird laut DGEM-Leitlinie nur für Patient:innen auf der ITS/IMC mit Indikation für eine (rein) parenterale Ernährung und ohne Multiorgandysfunktion bzw. renale oder hepatische Dysfunktion empfohlen, eine enterale Glutaminpharmakotherapie dagegen nicht [2]. Während in der DGEM-Leitlinie diesbezüglich keine Spiegelbestimmung empfohlen wird, gibt die ESPEN dagegen in einem Positionspapier Empfehlungen zur Spiegelbestimmung bei selektierten Patient:innen mit parenteraler Ernährung, kontinuierlicher Nierenersatztherapie, und Verbrennungen unter Einsatz eines nicht in Deutschland verfügbaren Point-of-Care-Analysegeräts [25]. Unter Beachtung der fachinformationskonformen Dosierung und der Gegenanzeigen und nicht zuletzt auch aufgrund des hohen methodischen Aufwands halten wir jedoch eine Messung der Plasmaglutaminspiegel nach wie vor für nicht erforderlich.

#### Empfehlung

- Der Plasma-Cu-Spiegel sollte bei Patient:innen mit schweren Verbrennungen, mechanischer Nierenersatztherapie > 2 Wochen und langfristiger PE bestimmt werden.
- Der Plasmazinkspiegel sollte bei Patient:innen mit erhöhten GI-Verlusten und Verbrennungen regelmäßig, bei langfristiger PE alle 6–12 Monate bestimmt werden.
- Der Vitamin-D-Spiegel (Serum-25[OH]D) kann bei allen Patient:innen mit Risiko für Mangelernährung oder Vitamin-D-Mangel bei Aufnahme auf die ITS/IMC bestimmt werden.
- Der Selenspiegel kann bei langfristiger PE bestimmt werden.
- Eine Spiegelbestimmung anderer Mikronährstoffe kann nicht empfohlen werden.
- Eine Spiegelbestimmung von Glutamin kann nicht empfohlen werden.

## 5. Bestimmung des Energieumsatzes

Zur Bestimmung des Ruheenergieumsatzes (REU) und somit des kalorischen Ziels im Rahmen der MNT sollte die indirekte Kalorimetrie eingesetzt werden, so lautet die einstimmige Empfehlung aller aktuellen intensivmedizinischen Leitlinien (inter‑)nationaler Ernährungsgesellschaften [2, 34, 53, 54].

Alter, BMI und Geschlecht sind unabhängige Determinanten des REU [55]. Der REU kritisch Kranker ist auch nicht konstant, sondern dynamisch und weist je nach Phase bzw. Art und Schwere der kritischen Erkrankung (z. B. Sepsis oder septischer Schock, Verbrennung oder Trauma) hohe intra- und interindividuelle Schwankungen auf. Bei beatmeten COVID-19-Patient:innen zeigte sich in einer kleinen Fallstudie ein persistierender Hypermetabolismus mit phasenweiser Steigerung des Ruheenergieumsatzes [55]. Eine solche Dynamik lässt sich mit anthropometrischen Schätzformeln zur Kalkulation des REU nicht abbilden. Allein die Messung mittels indirekter Kalorimetrie ermöglicht es, diese Schwankungen im REU korrekt zu erfassen. Die Methodik der indirekten Kalorimetrie erlaubt zudem durch die gleichzeitige Messung von CO_2_-Produktion (VCO_2_) und O_2_-Verbrauch (VO_2_) sowie die Berechnung des respiratorischen Quotienten (RQ) eine grobe Abschätzung der vorwiegend oxidativ utilisierten Energieträger (Fettsäuren vs. Kohlenhydrate; Tab. 3).

**Table 3:** 

Spirometrische Messung der inspiratorischen und exspiratorischen O_2_- und CO_2_-Konzentration und des exspirierten Gasvolumens/-flusses pro Minute
Darüber Kalkulation O_2_-Verbrauch (VO_2_) und CO_2_-Produktion (VCO_2_) in l/min VO_2_ = (V_i_ × F_i_O_2_) − (V_e_ × F_e_O_2_) und VCO_2_ = (V_e_ × F_e_CO_2_) − (V_i_ × F_i_CO_2_)
Da es technisch schwierig ist, den geringen Volumenunterschied zwischen Inspirations- und Exspirationsluft zu messen, wird V_i_ in der Regel anhand der Haldane-Transformation (N_2_ im in- und exspirierten Gas als konstant angenommen) kalkuliert V_i_ = [F_e_N_2_/F_i_N_2_] × V_e_ F_e_N_2_ = (1 − F_e_O_2_ − F_e_CO_2_) F_i_N_2_ = (1 − F_i_O_2_ − F_i_CO_2_) Wenn ein F_i_CO_2_ von 0,03–0,05 % ignoriert wird, dann: VO_2_ = [(1 − F_e_O_2_ − F_e_CO_2_) × (F_i_O_2_ − F_e_O_2_) × V_e_]/(1 − F_i_O_2_)
Modifizierte Weir-Formel für Ruheenergieumsatz (REU, kcal/Tag) = [3,9 (VO_2_) + 1,1 (VCO_2_)] × 1,44 Respiratorischer Quotient (RQ) = VCO_2_/VO_2_ RQ von 1,0; 0,8; und 0,7 ≃ Glukose‑, Protein- und Fettoxidation Physiologischer Range 0,67–1,3 (Qualitätsindikator der Messung)
Messtechnische bzw. patient:innenseitige Limitationen, die zu verfälschten Messergebnissen führen können, u. a. – Stickstoffmonoxid, Inhalationsanästhetika (aktive Befeuchtung möglich), – F_i_O_2_ > 0,6–0,7; variierend je nach Herstellerangaben – Leckagen im Beatmungssystem, extrakorporale Membranoxygenierung – Möglicher Messunterschied mit/ohne kontinuierliche Nierenersatztherapie unter Zitratantikoagulation (O_2_-CO_2_-Austausch, Austausch kalorienhaltigen Zitrats)

*F*_*i*_*N*_*2*_*/F*_e_*N*_*2*_ inspiratorische/exspiratorische Stickstoffkonzentration, *F*_*i*_*O*_*2*_*/F*_*i*_*CO*_*2*_ inspiratorische Sauerstoff‑/Kohlendioxidkonzentration, *F*_e_*O*_*2*_*/F*_e_*CO*_*2*_ exspiratorische Sauerstoff‑/Kohlendioxidkonzentration, *PEEP* positiver endexspiratorischer Druck, *RQ* respiratorischer Quotient, *VCO*_*2*_ Kohlendioxidproduktion, *VO*_*2*_ Sauerstoffverbrauch, *V*_*i*_ inspiratorisches Volumen, *V*_e_ exspiratorisches Volumen

Der Nutzen einer individuellen Steuerung der MNT anhand des gemessenen REU konnte allerdings wissenschaftlich bisher nicht eindeutig belegt werden. So divergieren auch die Ergebnisse aktueller Metaanalysen bez. des Effekts auf klinische Endpunkte bei Einsatz der indirekten Kalorimetrie in der Akutphase der kritischen Erkrankung [56–58]. In allen Studien, die bisher die klinische Relevanz der indirekten Kalorimetrie untersuchten, erfolgte zwar eine Individualisierung anhand des REU, jedoch nicht anhand der individuellen metabolischen Toleranz. Diese Limitierung könnte die Vorteile der MNT-Steuerung nach gemessenem REU aufgehoben haben. Besonders zu betonen ist also, dass speziell in der Akutphase einer kritischen Erkrankung der REU nicht als das in jedem Fall anzustrebende kalorische Ziel interpretiert werden sollte (exogene Energiezufuhr als Ersatz von endogen utilisierten Substraten im Verhältnis 1:1). Die Energiezufuhr sollte sich somit neben dem REU auch an den Möglichkeiten des Organismus orientieren, exogene Nährstoffe verwerten zu können. Daher wird – je nach individueller metabolischer Toleranz – oft nur ein gewisser Prozentsatz des tatsächlich gemessenen REU (30–50 %, 50–70 % oder 70–100 %) konkret als Kalorien zugeführt werden können.

Von einem besonderen Nutzen der indirekten Kalorimetrie ist auch bei Adipositas (BMI > 30 kg/m^2^) auszugehen, da hier die alternative Bestimmung des REU mittels Schätzformel einer „doppelten Ungenauigkeit“ unterliegt, indem ggf. zusätzlich eine Näherungsformel zur Bestimmung des Idealgewichts/adjustierten Körpergewichts zur Anwendung kommen muss [2, 34, 60].

Nur wenn eine indirekte Kalorimetrie nicht zur Verfügung steht oder messtechnische bzw. patient:innenseitige Limitationen bez. des Einsatzes der indirekten Kalorimetrie vorliegen (Tab. 3), wird in der DGEM-Leitlinie eine körpergewichts- bzw. BMI-bezogene Schätzung des Energieumsatzes empfohlen [2].

Tab. 4 zeigt eine Checkliste zur Durchführung der kalorimetrischen Messung in der klinischen Praxis. Bei der aktuellen Generation von Kalorimetriegeräten sind einige Nachteile älterer Geräte, insbesondere die Notwendigkeit einer aufwändigen Kalibrierung und der erhebliche Bedienaufwand bei nur mangelnder Genauigkeit eliminiert. Auch sind diese neueren Geräte nicht mehr nur als „Stand-alone“-Systeme verfügbar, sondern können in existierende Monitoringsysteme integriert werden.

**Table 4:** 

	Invasive Beatmung	Spontanatmung
Setup	✔ Korrekte Konnektion der Patient:innen an das System gemäß Herstellerangaben für das jeweilige System ✔ Eingabe notwendiger Patient:innendaten, z. B. Alter, Größe, Gewicht, und Autokalibrierung (Dauer in der Regel < 5 min, bis die Messungen begonnen werden können)	
1. Messintervall	✔ Idealerweise täglich ✔ Nach relevanten Änderungen des klinischen Zustands	
2. Respiration	✔ F_i_O_2_ < 0,7 ✔ Beatmungsdrücke dokumentieren zum Vergleich bei repetitiven Messungen ✔ Keine inhalative Stickstoffmonoxid-Therapie oder inhalative Sedierung mit Narkosegasen ✔ Ausschluss von Luftleckagen wie Thoraxdrainage etc., Cuff dicht?	✔ Dichtsitzende Maske? ✔ Toleranz/Vigilanz?
3. Agitation	Bei agitierten Patient:innen keine valide Messung möglich	
4. Maßnahmen sollten nicht innerhalb von < 60 min vor Messung erfolgen	✔ ±1 °C Temperaturänderung? Herzfrequenz? ✔ Relevante Medikationsdosisänderung (β-Blocker, Katecholamine) ✔ Physiotherapie/Mobilisation? ✔ Beatmungsparameter	
6. Ernährung	✔ Patient:in zum Zeitpunkt der Messung nüchtern oder ernährt? ✔ Wenn ernährt, passt die Zusammensetzung Makronährstoffe in Relation zum gemessenen RQ?	
7. Umgebung inkl. extrakorporale Therapie	✔ Körperposition zum Messzeitpunkt dokumentieren zum Vergleich bei repetitiven Messungen im Verlauf ✔ Keine extrakorporale Membranoxygenierung ✔ Nierenersatztherapie ja/nein im Vergleich zu Vormessung?	
8. Messqualität	✔ Valide Daten > 10 min Messdauer, metabolisches Äquilibrium: Variationskoeffizient < 5 % für VO_2_/VCO_2_ für 5 min! ✔ RQ plausibel (RQ > 1,3 oder < 0,67 = invalide Messung)?	
9. Desinfektion	Einmalartikel gemäß Hygiene verwerfen, Modul etc. reinigen	

*F*_*i*_*O*_*2*_*/F*_*i*_*CO*_*2*_ inspiratorische Sauerstoff‑/Kohlendioxidkonzentration, *PEEP* positiver endexspiratorischer Druck, *RQ* respiratorischer Quotient, *VCO*_*2*_ Kohlendioxidproduktion, *VO*_*2*_ Sauerstoffverbrauch

### Empfehlung

Zur Bestimmung des Ruheenergieumsatzes sollte die indirekte Kalorimetrie idealerweise täglich, also ab dem Zeitpunkt der ITS-/IMC-Aufnahme, eingesetzt werden (unter Beachtung der messtechnischen Limitationen) insbesondere bei kritisch kranken Patient:innen mit

- vorbestehender Malnutrition
- einer voraussichtlichen Behandlungsdauer ≥ 7 Tage auf der ITS/IMC
- bei Adipositas (BMI > 30 kg/m^2^).

## 6. Therapiesteuerung anhand der metabolischen Toleranz (Ausmaß des Insulinbedarfs)

**Figure 1:**
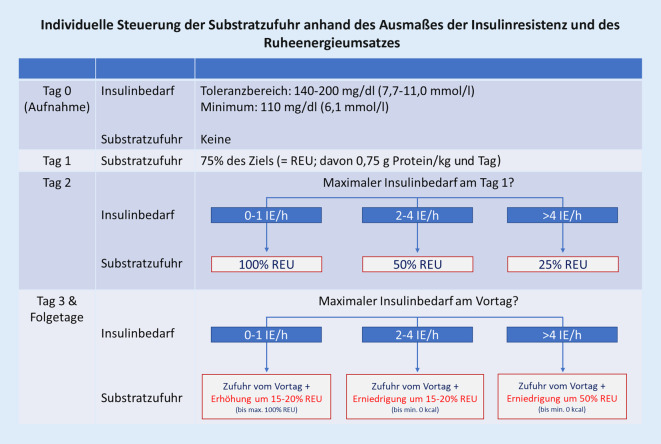

Die DGEM-Leitlinie [2] empfiehlt eine Steuerung der MNT in allen Phasen der kritischen Erkrankung entsprechend des REU und der individuellen Fähigkeit, exogene Substrate verwerten zu können („metabolische Toleranz“). Als Surrogatmarker für Substratintoleranzen dient das Ausmaß der Insulinresistenz (unter der Voraussetzung normaler Magnesiumspiegel, da ein entsprechender Mangel intrinsisch die Insulinresistenz erhöhen kann). Kommt es in der Akutphase an einem bestimmten Tag ein- oder mehrmalig zu einem exzessiven Insulinbedarf (> 4 IE/h zur Aufrechterhaltung einer Zielblutglukosekonzentration < 200 mg/dl bzw. < 11 mmol/l [Diabetes II: < 250 mg/dl bzw. < 13,9 mmol/l]), sollte eine Reduktion der aktuell praktizierten exogenen Substratzufuhr durchgeführt werden. Das Ausmaß der Reduktion richtet sich nach der Höhe des individuellen Insulinbedarfs. Optimale Schwellen sind nicht bekannt, die Empfehlungen orientieren sich an den Beobachtungen in der Praxis (speziell am durchschnittlichen Insulinbedarf). Bei nicht beherrschbarer Intoleranz kann eine komplette Unterbrechung der Kalorienzufuhr bzw. eine dann notwendige weitere Steigerung der Insulinzufuhr nötig sein, um die Blutzuckerkonzentration in den Toleranzbereich zu bringen. Abb. 1 zeigt ein entsprechendes Schema zur Steuerung der Substratzufuhr in Abhängigkeit vom kalorischen Ziel (= REU). Es wird darauf hingewiesen, dass die Empfehlungen für Patient:innen mit vorbestehendem Diabetes mellitus Typ II aus Sicht des Toleranzbereichs für die Blutzuckerkonzentration [62] eine Modifikation der ursprünglichen DGEM-Leitlinie [2] darstellen. Eine Extrapolation auf Patient:innen mit Diabetes mellitus Typ I ist bisher nicht möglich.

Sind sowohl der Insulinbedarf erhöht als auch die Phosphatkonzentration erniedrigt (vgl. oben), dominiert die Steuerung der Substratzufuhr anhand des Parameters, der die stärkste absolute Veränderung der Zufuhrrate erfordert. Bei normaler Phosphatkonzentration dominiert die Steuerung der Substratzufuhr anhand des Insulinbedarfs.

## 7. Vorgehen in der Praxis

Zusammenfassend sollte bei Patient:innen mit vorbestehender Malnutrition, einem voraussichtlichen Aufenthalt ≥ 7 Tagen auf der ITS/IMC, und/oder bei adipösen Patient:innen (BMI > 30 kg/m^2^) die Indikation für eine serielle indirekte Kalorimetrie idealerweise täglich ab dem Zeitpunkt der Aufnahme gestellt werden (unter Beachtung der messtechnischen Limitationen). Ferner durchgeführt werden sollte eine Analyse des Insulinbedarfs vom Vortag und eine Bestimmung der Plasmaphosphatkonzentration.

Die Bestimmung von Mikronährstoffkonzentrationen und ggf. daraus abzuleitende therapeutische Maßnahmen orientieren sich an den Empfehlungen in Tab. 2 und erstrecken sich nicht auf alle kritisch Kranken.

Offen ist die Frage, inwieweit bei Risikopatient:innen mit einer personalisierten und optimierten MNT ein Muskelabbau komplett verhindert (und nicht nur minimiert) werden kann, um so das Behandlungsergebnis zu verbessern. Unklar ist bis heute ebenfalls die Optimierung der MNT im Hinblick auf immunologische und reparative Funktionen [2]. Grundvoraussetzung einer effektiven MNT ist immer, durch kausale Therapie die Signalketten zu unterbrechen, die die Katabolie von Seite des inflammatorischen/infektiösen Stimulus her auslösen [2].

## References

[1] Weimann A, Hartl WH, Adolph M (2022). Erfassung und apparatives Monitoring des Ernährungsstatus von Patient*innen auf der Intensiv- und Intermediate Care Station. Med Klin Intensivmed Notfmed.

[2] Elke G, Hartl WH, Kreymann KG, Adolph M, Felbinger TW, Graf T, de Heer G, Heller A, Kampa U, Mayer K, Muhl E, Niemann B, Rümelin A, Steiner S, Stoppe C, Weimann A, Bischoff SC (2018). DGEM-Leitlinie: „Klinische Ernährung in der Intensivmedizin“. Aktuel Ernahrungsmed.

[3] Waydhas C, Riessen R, Markewitz A, Hoffmann E, Frey L, Böttiger BW, Brenner S, Brenner T, Deffner T, Deininger M, Janssen U, Kluge S, Marx G, Schwab S, Unterberg A, Walcher F, van den Hooven T (2022). Empfehlungen zur Struktur und Ausstattung von Intensivstationen 2022 – Kurzfassung. DIVI.

[4] Intermediate Care Station Empfehlungen zur Ausstattung und Struktur. https://www.divi.de/empfehlungen/publikationen/viewdocument/103/empfehlungen-zur-struktur-von-imc-stationen-kurzversion. Zugegriffen: 1. Dez. 2022 10.1007/s00063-017-0369-7 29116361

[5] Cederholm T, Jensen GL, Correia M, Gonzalez MC, Fukushima R, Higashiguchi T, Baptista G, Barazzoni R, Blaauw R, Coats A, Crivelli A, Evans DC, Gramlich L, Fuchs-Tarlovsky V, Keller H, Llido L, Malone A, Mogensen KM, Morley JE, Muscaritoli M, Nyulasi I, Pirlich M, Pisprasert V, de van der Schueren MAE, Siltharm S, Singer P, Tappenden K, Velasco N, Waitzberg D, Yamwong P, Yu J, Van Gossum A, Compher C (2019). GLIM criteria for the diagnosis of malnutrition—A consensus report from the global clinical nutrition community. Clin Nutr.

[6] Kondrup J (2014). Nutritional-risk scoring systems in the intensive care unit. Curr Opin Clin Nutr Metab Care.

[7] Evans DC, Corkins MR, Malone A, Miller S, Mogensen KM, Guenter P, Jensen GL, Committee AM (2021). The use of visceral proteins as nutrition markers: an ASPEN position paper. Nutr Clin Pract.

[8] Jensen GL, Cederholm T, Correia M, Gonzalez MC, Fukushima R, Higashiguchi T, de Baptista GA, Barazzoni R, Blaauw R, Coats AJS, Crivelli A, Evans DC, Gramlich L, Fuchs-Tarlovsky V, Keller H, Llido L, Malone A, Mogensen KM, Morley JE, Muscaritoli M, Nyulasi I, Pirlich M, Pisprasert V, de van der Schueren M, Siltharm S, Singer P, Tappenden KA, Velasco N, Waitzberg DL, Yamwong P, Yu J, Compher C, Van Gossum A (2019). GLIM criteria for the diagnosis of malnutrition: a consensus report from the global clinical nutrition community. JPEN J Parenter Enteral Nutr.

[9] Sharma S, Hashmi MF, Castro D (2021). Hypophosphatemia. StatPearls.

[10] Doig GS, Simpson F, Heighes PT, Bellomo R, Chesher D, Caterson ID, Reade MC, Harrigan PW (2015). Restricted versus continued standard caloric intake during the management of refeeding syndrome in critically ill adults: a randomised, parallel-group, multicentre, single-blind controlled trial. Lancet Respir Med.

[11] Berger MM, Appelberg O, Reintam-Blaser A, Ichai C, Joannes-Boyau O, Casaer M, Schaller SJ, Gunst J, Starkopf J (2021). Prevalence of hypophosphatemia in the ICU—Results of an international one-day point prevalence survey. Clin Nutr.

[12] Cohen J, Kogan A, Sahar G, Lev S, Vidne B, Singer P (2004). Hypophosphatemia following open heart surgery: incidence and consequences. Eur J Cardiothorac Surg.

[13] Reintam Blaser A, Gunst J, Ichai C, Casaer MP, Benstoem C, Besch G, Dauger S, Fruhwald SM, Hiesmayr M, Joannes-Boyau O, Malbrain M, Perez MH, Schaller SJ, de Man A, Starkopf J, Tamme K, Wernerman J, Berger MM (2021). Hypophosphatemia in critically ill adults and children—A systematic review. Clin Nutr.

[14] Zazzo JF, Troche G, Ruel P, Maintenant J (1995). High incidence of hypophosphatemia in surgical intensive care patients: efficacy of phosphorus therapy on myocardial function. Intensive Care Med.

[15] Di Mario F, Regolisti G, Greco P, Maccari C, Superchi E, Morabito S, Pistolesi V, Fiaccadori E (2021). Prevention of hypomagnesemia in critically ill patients with acute kidney injury on continuous kidney replacement therapy: the role of early supplementation and close monitoring. J Nephrol.

[16] Hartl WH, Parhofer KG, Kuppinger D, Rittler P (2013). S3-Leitlinie der Deutschen Gesellschaft für Ernährungsmedizin (DGEM) in Zusammenarbeit mit der GESKES und der AKE: Besonderheiten der Überwachung bei künstlicher Ernährung. Aktuel Ernahrungsmed.

[17] Bohe J, Abidi H, Brunot V, Klich A, Klouche K, Sedillot N, Tchenio X, Quenot JP, Roudaut JB, Mottard N, Thiolliere F, Dellamonica J, Wallet F, Souweine B, Lautrette A, Preiser JC, Timsit JF, Vacheron CH, Ait Hssain A, Maucort-Boulch D (2021). Individualised versus conventional glucose control in critically-ill patients: the CONTROLING study—A randomized clinical trial. Intensive Care Med.

[18] Deane AM, Plummer MP, Ali Abdelhamid Y (2022). Update on glucose control during and after critical illness. Curr Opin Crit Care.

[19] Poole AP, Finnis ME, Anstey J, Bellomo R, Bihari S, Biradar V, Doherty S, Eastwood G, Finfer S, French CJ, Heller S, Horowitz M, Kar P, Kruger PS, Maiden MJ, Martensson J, McArthur CJ, McGuinness SP, Secombe PJ, Tobin AE, Udy AA, Young PJ, Deane AM (2022). The effect of a liberal approach to glucose control in critically ill patients with type 2 diabetes: a multicenter, parallel-group, open-label randomized clinical trial. Am J Respir Crit Care Med.

[20] Laufs U, Parhofer KG, Ginsberg HN, Hegele RA (2020). Clinical review on triglycerides. Eur Heart J.

[21] Packard CJ, Boren J, Taskinen MR (2020). Causes and consequences of hypertriglyceridemia. Front Endocrinol (Lausanne).

[22] Saja MF, Cook HT, Ruseva MM, Szajna M, Pickering MC, Woollard KJ, Botto M (2018). A triglyceride-rich lipoprotein environment exacerbates renal injury in the accelerated nephrotoxic nephritis model. Clin Exp Immunol.

[23] Si M, Li D, Liu T, Cai Y, Yang J, Jiang L, Yu H (2022). Triglycerides as biomarker for predicting systemic lupus erythematosus related kidney injury of negative proteinuria. Biomolecules.

[24] Devaud JC, Berger MM, Pannatier A, Marques-Vidal P, Tappy L, Rodondi N, Chiolero R, Voirol P (2012). Hypertriglyceridemia: a potential side effect of propofol sedation in critical illness. Intensive Care Med.

[25] Berger MM, Reintam-Blaser A, Calder PC, Casaer M, Hiesmayr MJ, Mayer K, Montejo JC, Pichard C, Preiser JC, van Zanten ARH, Bischoff SC, Singer P (2019). Monitoring nutrition in the ICU. Clin Nutr.

[26] Mayer K, Klek S, Garcia-de-Lorenzo A, Rosenthal MD, Li A, Evans DC, Muscaritoli M, Martindale RG (2020). Lipid use in hospitalized adults requiring parenteral nutrition. JPEN J Parenter Enteral Nutr.

[27] Barreto EF, Kanderi T, DiCecco SR, Lopez-Ruiz A, Poyant JO, Mara KC, Heimgartner J, Gajic O, Rule AD, Nystrom EM, Kashani KB (2019). Sarcopenia index is a simple objective screening tool for malnutrition in the critically ill. JPEN J Parenter Enteral Nutr.

[28] Barreto EF, Poyant JO, Coville HH, Dierkhising RA, Kennedy CC, Gajic O, Nystrom EM, Takahashi N, Moynagh MR, Kashani KB (2019). Validation of the sarcopenia index to assess muscle mass in the critically ill: A novel application of kidney function markers. Clin Nutr.

[29] Calvani R, Picca A, Cesari M, Tosato M, Marini F, Manes-Gravina E, Bernabei R, Landi F, Marzetti E (2018). Biomarkers for sarcopenia: reductionism vs. complexity. Curr Protein Pept Sci.

[30] Haines RW, Zolfaghari P, Wan Y, Pearse RM, Puthucheary Z, Prowle JR (2019). Elevated urea-to-creatinine ratio provides a biochemical signature of muscle catabolism and persistent critical illness after major trauma. Intensive Care Med.

[31] Kashani KB, Frazee EN, Kukralova L, Sarvottam K, Herasevich V, Young PM, Kashyap R, Lieske JC (2017). Evaluating muscle mass by using markers of kidney function: development of the sarcopenia index. Crit Care Med.

[32] Page A, Flower L, Prowle J, Puthucheary Z (2021). Novel methods to identify and measure catabolism. Curr Opin Crit Care.

[33] Tosato M, Marzetti E, Cesari M, Savera G, Miller RR, Bernabei R, Landi F, Calvani R (2017). Measurement of muscle mass in sarcopenia: from imaging to biochemical markers. Aging Clin Exp Res.

[34] Singer P, Blaser AR, Berger MM, Alhazzani W, Calder PC, Casaer MP, Hiesmayr M, Mayer K, Montejo JC, Pichard C, Preiser JC, van Zanten ARH, Oczkowski S, Szczeklik W, Bischoff SC (2019). ESPEN guideline on clinical nutrition in the intensive care unit. Clin Nutr.

[35] Allingstrup MJ, Kondrup J, Wiis J, Claudius C, Pedersen UG, Hein-Rasmussen R, Bjerregaard MR, Steensen M, Jensen TH, Lange T, Madsen MB, Moller MH, Perner A (2017). Early goal-directed nutrition versus standard of care in adult intensive care patients: the single-centre, randomised, outcome assessor-blinded EAT-ICU trial. Intensive Care Med.

[36] Dickerson RN, Tidwell AC, Minard G, Croce MA, Brown RO (2005). Predicting total urinary nitrogen excretion from urinary urea nitrogen excretion in multiple-trauma patients receiving specialized nutritional support. Nutrition.

[37] Deutsche Gesellschaft für Ernährung e. V. DACH-Referenzwerte. https://www.dge.de/wissenschaft/referenzwerte/gesamt/. Zugegriffen: 1. Dez. 2022

[38] Berger MM, Shenkin A, Amrein K, Augsburger M, Biesalski HK, Bischoff SC, Casaer MP, Gundogan K, Lepp HL, de Man AME, Muscogiuri G, Pietka M, Pironi L, Rezzi S, Schweinlin A, Cuerda C (2022). ESPEN micronutrient guideline. Clin Nutr.

[39] Berger MM, Ben-Hamouda N (2020). Trace element and vitamin deficiency: quantum medicine or essential prescription?. Curr Opin Crit Care.

[40] Estensen K, Shekar K, Robins E, McDonald C, Barnett AG, Fraser JF (2014). Macro- and micronutrient disposition in an ex vivo model of extracorporeal membrane oxygenation. Intensive Care Med Exp.

[41] Fah M, Van Althuis LE, Ohnuma T, Winthrop HM, Haines KL, Williams DGA, Krishnamoorthy V, Raghunathan K, Wischmeyer PE (2022). Micronutrient deficiencies in critically ill patients receiving continuous renal replacement therapy. Clin Nutr ESPEN.

[42] Gundogan K, Yucesoy FS, Ozer NT, Temel S, Sahin S, Sahin GG, Sungur M, Esmaoglu A, Talih T, Yazici C, Griffith DP, Ziegler TR (2022). Serum micronutrient levels in critically ill patients receiving continuous renal replacement therapy: A prospective, observational study. JPEN J Parenter Enteral Nutr.

[43] Lindberg BR, Videm V, Dahl T, Sorensen G, Fiane AE, Thiara AS (2020). Influence of the ECMO circuit on the concentration of nutritional supplements. Sci Rep.

[44] Schneider AG, Picard W, Honore PM, Dewitte A, Mesli S, Redonnet-Vernhet I, Fleureau C, Ouattara A, Berger MM, Joannes-Boyau O (2021). Amino acids and vitamins status during continuous renal replacement therapy: An ancillary prospective observational study of a randomised control trial. Anaesth Crit Care Pain Med.

[45] Duncan A, Talwar D, McMillan DC, Stefanowicz F, O’Reilly DS (2012). Quantitative data on the magnitude of the systemic inflammatory response and its effect on micronutrient status based on plasma measurements. Am J Clin Nutr.

[46] Brodin O, Hackler J, Misra S, Wendt S, Sun Q, Laaf E, Stoppe C, Bjornstedt M, Schomburg L (2020). Selenoprotein P as biomarker of selenium status in clinical trials with therapeutic dosages of selenite. Nutrients.

[47] Sedhai YR, Shrestha DB, Budhathoki P, Jha V, Mandal SK, Karki S, Baniya R, Cable CA, Kashiouris MG (2021). Effect of thiamine supplementation in critically ill patients: A systematic review and meta-analysis. J Crit Care.

[48] Lamontagne F, Masse MH, Menard J, Sprague S, Pinto R, Heyland DK, Cook DJ, Battista MC, Day AG, Guyatt GH, Kanji S, Parke R, McGuinness SP, Tirupakuzhi Vijayaraghavan BK, Annane D, Cohen D, Arabi YM, Bolduc B, Marinoff N, Rochwerg B, Millen T, Meade MO, Hand L, Watpool I, Porteous R, Young PJ, D’Aragon F, Belley-Cote EP, Carbonneau E, Clarke F, Maslove DM, Hunt M, Chasse M, Lebrasseur M, Lauzier F, Mehta S, Quiroz-Martinez H, Rewa OG, Charbonney E, Seely AJE, Kutsogiannis DJ, LeBlanc R, Mekontso-Dessap A, Mele TS, Turgeon AF, Wood G, Kohli SS, Shahin J, Twardowski P, Adhikari NKJ (2022). Intravenous vitamin C in adults with sepsis in the intensive care unit. N Engl J Med.

[49] Agarwal A, Basmaji J, Fernando S, Zhou Ge F, Xiao Y, Faisal H, Honarmand K, Hylands M, Lau V, Lewis K, Couban R, Lamontagne F, Adhikari N (2022). Parenteral vitamin C in patients with severe infection: a systematic review. NEJM Evid.

[50] Menger J, Lee ZY, Notz Q, Wallqvist J, Hasan MS, Elke G, Dworschak M, Meybohm P, Heyland DK, Stoppe C (2022). Administration of vitamin D and its metabolites in critically ill adult patients: an updated systematic review with meta-analysis of randomized controlled trials. Crit Care.

[51] Gudivada KK, Kumar A, Sriram K, Baby J, Shariff M, Sampath S, Sivakoti S, Krishna B (2022). Antioxidant micronutrient supplements for adult critically ill patients: A bayesian multiple treatment comparisons meta-analysis. Clin Nutr ESPEN.

[52] Blaauw R, Osland E, Sriram K, Ali A, Allard JP, Ball P, Chan LN, Jurewitsch B, Logan Coughlin K, Manzanares W, Menendez AM, Mutiara R, Rosenfeld R, Sioson M, Visser J, Berger MM (2019). Parenteral provision of micronutrients to adult patients: an expert consensus paper. JPEN J Parenter Enteral Nutr.

[53] Compher C, Bingham AL, McCall M, Patel J, Rice TW, Braunschweig C, McKeever L (2022). Guidelines for the provision of nutrition support therapy in the adult critically ill patient: The American Society for Parenteral and Enteral Nutrition. JPEN J Parenter Enteral Nutr.

[54] Reintam Blaser A, Starkopf J, Alhazzani W, Berger MM, Casaer MP, Deane AM, Fruhwald S, Hiesmayr M, Ichai C, Jakob SM, Loudet CI, Malbrain ML, Montejo Gonzalez JC, Paugam-Burtz C, Poeze M, Preiser JC, Singer P, van Zanten AR, De Waele J, Wendon J, Wernerman J, Whitehouse T, Wilmer A, Oudemans-van Straaten HM (2017). Early enteral nutrition in critically ill patients: ESICM clinical practice guidelines. Intensive Care Med.

[55] Whittle J, Molinger J, MacLeod D, Haines K, Wischmeyer PE (2020). Persistent hypermetabolism and longitudinal energy expenditure in critically ill patients with COVID-19. Crit Care.

[56] Duan JY, Zheng WH, Zhou H, Xu Y, Huang HB (2021). Energy delivery guided by indirect calorimetry in critically ill patients: a systematic review and meta-analysis. Crit Care.

[57] Pertzov B, Bar-Yoseph H, Menndel Y, Bendavid I, Kagan I, Glass YD, Singer P (2021). The effect of indirect calorimetry guided isocaloric nutrition on mortality in critically ill patients—A systematic review and meta-analysis. Eur J Clin Nutr.

[58] Tatucu-Babet OA, Fetterplace K, Lambell K, Miller E, Deane AM, Ridley EJ (2020). Is energy delivery guided by indirect calorimetry associated with improved clinical outcomes in critically ill patients? A systematic review and meta-analysis. Nutr Metab Insights.

[59] Mtaweh H, Tuira L, Floh AA, Parshuram CS (2018). Indirect calorimetry: History, technology, and application. Front Pediatr.

[60] McClave SA, Taylor BE, Martindale RG, Warren MM, Johnson DR, Braunschweig C, McCarthy MS, Davanos E, Rice TW, Cresci GA, Gervasio JM, Sacks GS, Roberts PR, Compher C (2016). Guidelines for the provision and assessment of nutrition support therapy in the adult critically Ill patient: Society of Critical Care Medicine (SCCM) and American Society for Parenteral and Enteral Nutrition (A.S.P.E.N.). JPEN J Parenter Enteral Nutr.

[61] Oshima T, Berger MM, De Waele E, Guttormsen AB, Heidegger CP, Hiesmayr M, Singer P, Wernerman J, Pichard C (2017). Indirect calorimetry in nutritional therapy. A position paper by the ICALIC study group. Clin Nutr.

[62] Poole AP, Finnis ME, Anstey J, Bellomo R, Bihari S, Birardar V, Doherty S, Eastwood G, Finfer S, French CJ, Heller S, Horowitz M, Kar P, Kruger PS, Maiden MJ, Martensson J, McArthur CJ, McGuinness SP, Secombe PJ, Tobin AE, Udy AA, Young PJ, Deane AM (2022). The effect of a liberal approach to glucose control in critically ill patients with type 2 diabetes: a multicenter, parallel-group, open-label, randomized clinical trial. Am J Respir Crit Care Med.

